# Prevalence of Systemic Lupus Erythematosus in South Korea: An Administrative Database Study

**DOI:** 10.2188/jea.JE20120204

**Published:** 2014-07-05

**Authors:** Ji Hyeon Ju, Sang-Heon Yoon, Kwi Young Kang, In Je Kim, Seung-Ki Kwok, Sung-Hwan Park, Ho-Youn Kim, Won-Chul Lee, Chul-Soo Cho

**Affiliations:** 1 Division of Rheumatology, Department of Internal Medicine, College of Medicine, The Catholic University of Korea, Seoul, South Korea; 2 Department of Preventive Medicine, College of Medicine, The Catholic University of Korea, Seoul, South Korea; 3 Health Insurance Review and Assessment Service

**Keywords:** systemic lupus erythematosus, prevalence, epidemiology

## Abstract

**Background:**

Systemic lupus erythematosus (SLE) is a rare autoimmune disease for which a population-based survey on the prevalence of the disease in South Korea has not yet been conducted. Our goal was to estimate the nationwide prevalence of SLE.

**Methods:**

The International Classification of Diseases, Tenth Revision (ICD-10) code for SLE diagnosis—M32—was tentatively given when patients were suspected to have SLE before 2009. As such, the positive predictive value (PPV) of the M32 code shown in medical bills reflecting true SLE was uncertain. We attempted to estimate the prevalence of SLE in South Korea using national administrative database data from 2004–2006. We approximated the actual number of SLE patients by analyzing a list of SLE-coded patients provided by the National Health Insurance (NHI) and Health Insurance Review and Assessment Service. Prevalence was estimated by multiplying the PPV of the M32 diagnostic code by the number of patients receiving the code. The PPV was determined by three methods: direct investigation of the medical records of patients randomly selected from the SLE-coded patients list; assessment of all SLE patients treated at 56 selected hospitals in South Korea; and extrapolation from sub-groups at a single institute to the sub-groups of the national NHI data.

**Results:**

The estimated number of national SLE cases was between 9000 and 11 000, depending on the method of ascertainment, corresponding to a prevalence of 18.8–21.7 per 100 000 people.

**Conclusions:**

This is the first report of a nationwide prevalence survey of SLE in South Korea. National databases may serve as a resource for epidemiologic studies of rare autoimmune diseases like SLE.

## INTRODUCTION

Systemic lupus erythematosus (SLE) is an autoimmune disease involving multiple systems, including the nervous, circulatory, pulmonary, renal, and immune systems.^1^^,^^2^ SLE is prevalent among younger individuals, primarily those aged in their 20s to 40s, and is more frequent in women than men.^3^ SLE is often complicated with seizures, renal insufficiency, and anemia, which substantially reduce quality of life.^4^^,^^5^ In order to improve management of SLE, it is necessary to clearly understand the prevalence of the disease, including the clinical and socioeconomic characteristics of SLE patients.^6^^–^^8^ However, no population-based prevalence survey of SLE in South Korea has so far been conducted.

Cohort studies involving primary data collection have been used to estimate SLE prevalence, but such studies tend to be costly and time-consuming.^9^^,^^10^ Alternatively, population-based administrative databases, containing physician billing and insurance information, have received increased attention for their potential to provide epidemiologic information, particularly for rare conditions like SLE.^11^^–^^13^ Along with reduced costs and time requirements, administrative data also offer the advantage of simplicity in establishing and maintaining a population-based surveillance system. However, optimal methods for extracting information from these databases have yet to be determined.^14^^,^^15^ Researchers have therefore called for further studies regarding the usefulness of administrative sources such as physician claims databases and insurance databases.^8^^,^^16^^,^^17^ To this end, studies concerning the positive predictive value (PPV) of diagnoses based on administrative data are required, especially for complex conditions such as SLE.

Korea has a medical insurance system, in which all citizens are required to join the national public insurance system and pay monthly medical insurance premiums. The information from medical practices is then collected by the National Health Insurance (NHI) system. This delicate process is strictly governed by the Health Insurance Review and Assessment Service (HIRAS). Therefore, national epidemiological data can be easily collected by analyzing the NHI payment request data from HIRAS. However, this database had a minor problem to overcome. In the past, the International Classification of Diseases, Tenth Revision (ICD-10) code for SLE diagnosis, M32, was often given tentatively to suspected SLE patients before the year of 2009. As such, the PPV of the M32 diagnosis was uncertain before 2009. We tried to estimate the prevalence of SLE using the national database from 2004 to 2006, which used the M32 code (probable SLE diagnosis). We calculated the “raw” prevalence of SLE from NHI data and then verified the SLE-coded diagnosis as follows: 1) verification of randomly selected SLE-coded samples; 2) verification of all registered SLE-coded patients from more than 56 hospitals nationwide; and 3) application of a PPV, obtained from subgroups of a sample institute, to the NHI database. Then, the calculated PPV was applied to adjust the raw prevalence, thereby estimating the prevalence of SLE in South Korea.

Recently, the South Korean government enacted a law requiring physicians to make a definitive diagnosis to bestow better insurance benefits on only patients whose diagnosis is confirmed to be SLE. Since this measure was introduced, more accurate data on the prevalence of SLE diagnosis have become available.

## MATERIALS AND METHODS

### Data assessment and acquisition of HIRAS data

**Figure 1.**
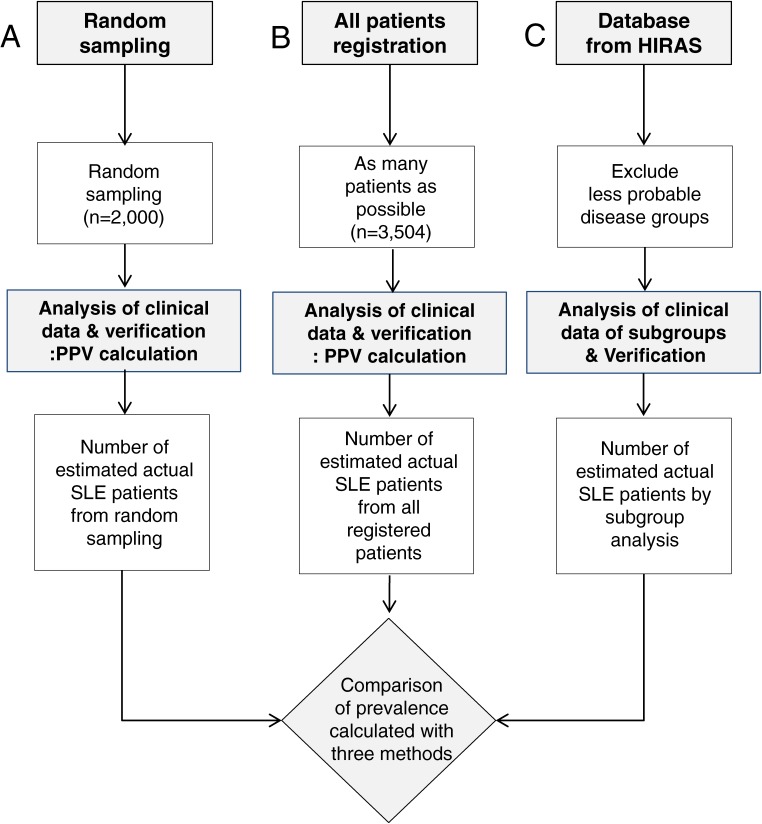
Three algorithmic approaches were adopted for the calculation of SLE prevalence, and the outcomes of each method were compared. The first method was to randomly select M32-coded patients in nationwide hospitals (A. Random sampling). Diagnostic accuracy was then evaluated by analysis of clinical data with verification on 2000 patients’ records. The second method was to register all possible patients obtained from rheumatologists in the Korean Rheumatism Association (B. All registered patients). A total of 3504 patients were registered, and their patient records were reviewed. Diagnostic accuracy was also delineated by analysis of clinical data in patient records. The third method was to exclude patients least likely to have SLE and include patients most likely SLE based on chronology of insurance claim issuing pattern (C. Interpreting HIRAS database).

SLE data were compiled from resources of HIRAS and provided to the research team for primary analysis. This process was officially permitted by the Korean Food and Drug Administration (KFDA) and HIRAS. The HIRAS statisticians compiled data on all patients from institutes nationwide who had been treated under the ICD-10 code of “M32” for 3 years from June 2004 to June 2007. Due to enforced privacy laws, detailed personal information was unable to be retrieved; instead, each patient was allocated an identification number. Data extraction and compilation processes are shown in Figure 1. The database contained information on service code (admission or outpatient clinic), year (billing year), age (year old), region (province), sex (male or female), hospital (private clinic, hospital, university hospital), department (internal medicine, dermatology, and so forth), admission (admission or not), and billing number.

### Analysis of HIRAS data

The HIRAS database is relatively credible and well-grounded for its use in managing the national statistical resources. Nevertheless, apparent limitations of the database stem from lack of accuracy of diagnosis, because the original purpose of the data was to manage national health insurance rather than for use in academic evaluation. To overcome this limitation, verification of the SLE-coded patients was necessary; if the PPV of the M32 code could be deduced, then the actual prevalence of SLE could be estimated. However, since the law restricts revelation of personal and institutional identifications, the diagnostic PPV of the M32 code could not be confirmed using this method.

### Verification processes of M32-coded group

Our initial plan was to randomly select a relatively small number of patients who were coded M32 and to review their medical records in order to validate their diagnosis. However, legal restriction regarding the protection of personal information mandated other alternatives, as follows: 1) verification after random sampling, which would randomly sort regional distribution, sex ratio, hospital grades, and age patterns of the original database; 2) verification after recruiting as many M32 patients as possible with the help of the 150 rheumatologists in the Korean Rheumatology Association; and 3) sub-grouping of M32 patients from the original SLE data and matching diagnostic PPVs between subgroups and Korean reference groups. Detailed explanations about randomization, data gathering, and analysis are presented in Figure 1.

### Verification of the patients who were randomly sampled

A total of 2000 patients were randomly selected and the validity of their diagnostic accuracy was analyzed (Figure 1A). We sought to investigate 15% of all M32-coded patients from each institute as an optimal proportion. Random sampling was done according to the ratio of regional differences and types of hospitals, with 868 patients (43.4%) in Seoul, 270 patients (13.5%) in Kyounggi province, 212 patients (10.6%) in Pusan, 126 patients (6.3%) in Daegu, and so on. In terms of hospital types, 1308 patients (65.4%) were in university hospitals, 358 patients (17.9%) were in general hospitals, 18 patients (0.9%) were in local hospitals, and 316 patients (15.8%) were in private clinics. Identifications of clinics were provided by the Korean Rheumatology Association. Before experienced rheumatologists were sent to each institute, randomization of patients was verified by a statistician. In addition, patient lists were sent to the institutes in advance so that their charts could be prepared for the verification process. National prevalence of SLE per 100 000 was based on the population in 2006 (http://kosis.nso.go.kr/Magazine/NEW/YD/VD00013.xls).

### Verification of all registered patients

After verification of randomly selected M32-coded patients, we decided to recruit as many M32-coded patients as possible to compensate for the limitations of random sampling (Figure 1B). Since SLE is relatively rare and sometimes fatal, most patients are referred to experienced rheumatologists. We sent official letters to every rheumatologist who was affiliated with the Korean Rheumatology Association. As a result, more than 160 rheumatologists participated in the survey, and a total of 3504 M32-coded patients were recruited for verification.

### Application of PPV of sample groups to the national database

**Figure 2.**
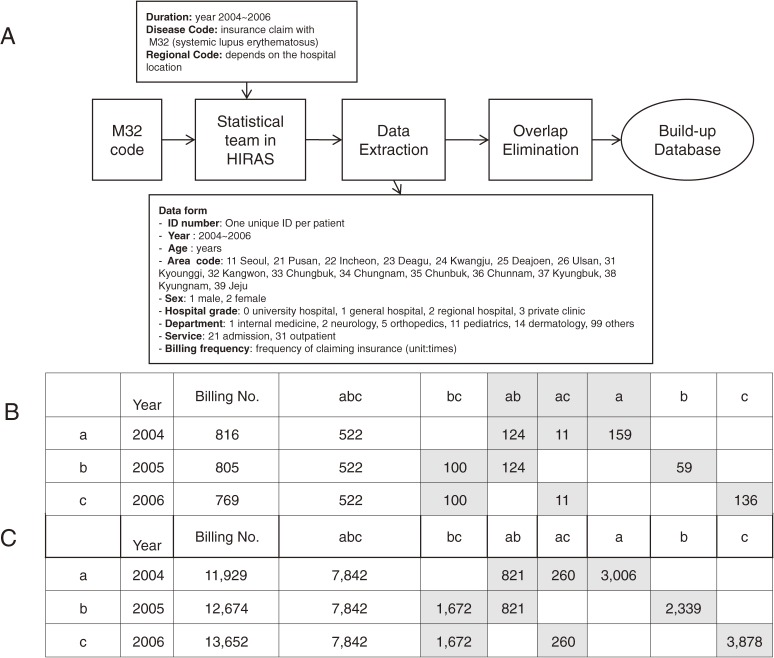
A. Database build-up process from government insurance data pool. Data were requested on M32-coded patients, including duration of disease, regional code, year of insurance claim, age, sex, hospital type (university hospital, general hospital, regional hospital, private clinic), department (internal medicine, neurology, pediatrics, etc), and type of treatment (admission, outpatient). Data were extracted from HIRAS and modified by statistical teams. Duplicate data were eliminated, and the finalized database was provided to our research team. B. The number of patients by year (2004, 2005, and 2006) when the medical records were issued in a single institute. “a, b, c” equals years 2004, 2005, and 2006, respectively. “abc” signifies the group of SLE patients with the diagnosis of SLE who were treated in a single institute in consecutive years of 2004, 2005, and 2006. “ac” signifies patients with the SLE diagnosis treated in 2004 and 2006. “a” signifies that the M32 code was used for only one year, 2004. C. The number of patients according to the year 2004, 2005, and 2006 when the bills were issued. “a, b, c” equals the years 2004, 2005, and 2006, respectively. “abc” signifies the group of SLE patients whose M32-coded insurance billings were issued in consecutive years of 2004, 2005, and 2006. “ac” signifies patients with the SLE diagnosis billed in 2004 and 2006. “a” signifies that the M32 code was used for only one year, 2004, for insurance billing. SLE, systemic lupus erythematosus; HIRAS, Health Insurance Review and Assessment Service.

Patterns of insurance coverage, frequency of a patient’s visits, type of healthcare provider, billing, and age are significant factors in the accurate diagnosis of SLE (Figure 2). Most importantly, an annual clinic visit is essential for SLE patients. We categorized 7 groups of M32-coded patients from the database according to number of annual visits to a clinic over three consecutive years. In the Group “abc”, patients visited clinics for three consecutive years, indicating a high probability of a correct SLE diagnosis. Group “bc” and “c” meant that patients visited a clinic continuously till the time of this investigation for 2 years and 1 year, respectively. Diagnosis of SLE seemed less accurate in other groups such as group “ab” and “a”, whose patient visits were remote, suggesting much lower probability of SLE. We calculated the diagnostic PPV of 7 groups in our institute that were applied to the national database (Figure 1C).

### Application of estimated prevalence of SLE to isolated regional database

We finally figured out SLE prevalence via three different methods. To verify whether this estimate is realistic or not, we compared this estimate with actual local prevalence in an isolated area. We accessed the database of Jeju Island, where two hospitals usually take care of SLE patients. We analyzed the data of these two hospitals and calculated the PPV of patients with an M32 code actually having SLE. We then calculated local prevalence on the island by the number of diagnosed patients multiplied by PPV and divided by the entire island population. This local prevalence was compared with the estimate from the national database.

### The pilot study

The pilot study was conducted in 7 hospitals affiliated with our institute. An initial data collection form for analysis was prepared and verified in the pilot study; however, preparing the data collection form was arduous and time-consuming. Even after careful evaluation of the medical record, the retrieved data were insufficient for the survey. Hence, a simple data collection form to register minimal yet essential SLE information was created. In the process of generating the data collection form, patients’ social security numbers were partially obscured to protect personal information and privacy.

### The data collection form and definition of SLE

The modified, simple data collection form solicited 11 types of patient information, which were the 1982 revised criteria of SLE^1^: presence of malar rash, discoid rash, photosensitivity, oral ulcer, arthritis, serositis, renal disorder, hematologic disorder, neurologic disorder, immunologic disorder, and positive fluorescent anti-nuclear antibody (FANA) test. Diagnosis of SLE was considered to be confirmed when 4 out of 11 criteria were satisfied.

### Statistical analysis

We had problems calculating the confidence intervals (CIs) shown in Table. The 95% CI should be obtained from prevalence data itself. However, our research environment prevented us from accessing full sets of data. During the estimation of SLE prevalence, we chose to calculate CIs of PPV and then multiplied by total population of M32-coded.

$$\begin{array}{l} \text{95}\%\mathrm{CI of fraction is}\\ \left[p-1.96*\mathrm{sqrt}\{p(1-p)/n\},p+1.96*\mathrm{sqrt}\{p(1-p)/n\}\right] \end{array}$$

where “p” is “PPV,” derived from the investigation of medical records of patients, and “n” is the number of patients whose medical records were reviewed.

**Table.** Estimated SLE prevalence according to three different ascertainment methods

Algorithms	Random sampling	All registered patients	Subgroup analysis
Estimated patients (95% CI)	9167 (8886–9447)	9533 (9326–9741)	10 633
Prevalence per 100 000 people (95% CI)	18.8 (18.2–19.3)	19.5 (19.1–19.9)	21.7

SLE, systemic lupus erythematosus; CI, confidence interval.

We thought that random sampling and the method of analyzing all registered M32 patients were more direct means of estimating PPV and could be compared with other potential methods of subgroup analysis. All statistical analyses were weighted to the Korean population to provide nationally representative estimates. We used the mid-year estimated population from 2006, provided by National Statistical Office website (http://www.kosis.kr). SAS (version 8.12; SAS Institute, Cary, NC, USA) was used for random sampling. Results were analyzed with SAS and Excel (version 2003; Microsoft Corporation, Redmond, WA, USA).

## RESULTS

### Basic characteristics of database subjects

**Figure 3.**
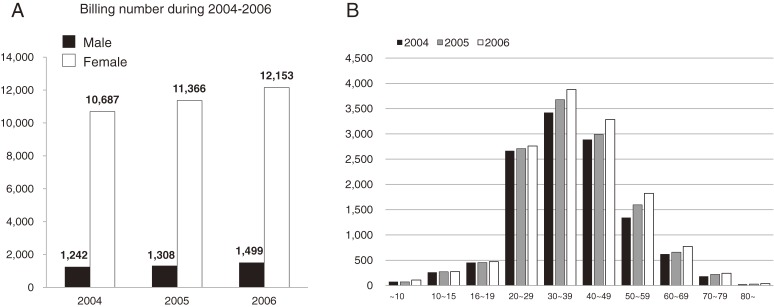
A. Annual increase in insurance claims with the M32 code in South Korea. Male to female ratio is about 10 to 1. The number of male and female patients increased each year. B. The peak prevalence is located in the age group of 30- to 39-year-olds. The population demonstrates a normal distribution around the peak.

According to the NHI database, the number of insurance claims with the M32 code incrementally increased from 2004 to 2006. Specifically, female patients increased by 1000 annually, while male patients increased by 100 annually (Figure 3A). The female-to-male ratio was 89.4:10.6. The 30- to 39-year-old age group was the most affected, followed by 40–49, 20–29, and 50–59 (Figure 3B). Korean SLE patients were managed in university hospitals (70%), general hospitals (19%), private clinics (18%), and regional hospitals (1%). Over 90% of SLE patients visited a rheumatology clinic, while approximately 3% of SLE patients went to either dermatology clinics or pediatric clinics.

### Diagnostic PPV calculated in patients with SLE-coded by randomization

As described in Figure 1A, we randomly selected 100 patients from private clinics, 400 patients from 13 general hospitals, and 1500 patients from 30 university hospitals in order to calculate the PPV of diagnosis in each of these settings. The PPV of diagnosis in private clinics was 0.65, while diagnostic PPVs in general hospitals and university hospitals were 0.70 and 0.76, respectively. Calculation of the prevalence was performed using the following equation:

$$\begin{array}{l} Presumed\;number\;of\;SLE\;patients\\ =Total\;number\;of\;M32\;insurance\;claims*PPV \end{array}$$

where the total number of insurance claims concerning M32 was 13 652, and the PPV was the actual number patients who meet the criteria divided by 2000. Among the random sample of SLE patients, the calculated PPV was 0.6715 (95% CI 0.6509–0.6920), resulting in a prevalence estimate of 9167 (95% CI 8886–9447).

### Diagnostic PPV calculated among SLE-coded patients from all institutes which participated in nationwide survey

As displayed in Figure 1B, every accessible M32-coded patient was investigated by rheumatologists contacted by the Korean Rheumatism Association. Twenty-two hospitals participated in this survey and registered 3504 SLE patients. With the previously calculated PPV of 69.8% in this setting, the prevalence was deduced using the following equation:

$$\begin{array}{l} Presumed\;number\;of\;SLE\;patients\\ =Total\;number\;of\;M32\;insurance\;claims*PPV \end{array}$$

where the total number of insurance claims concerning M32 was 13 652, and the PPV was the actual number of patients who meet the criteria divided by 3504. Among all participants seen by study rheumatologists, the PPV was 0.6983 (95% CI 0.6831–0.7135), resulting in an estimated prevalence of 9533 (95% CI 9326–9741).

### Diagnostic PPV of a single institute applied to the national database after subgroup analysis

As displayed in Figure 1C, a single institute, in which patient data could be investigated thoroughly, was chosen for sub-grouping according to clinic visitation patterns for the past 3 years (Figure 2B). We then calculated PPVs for each subgroup. Sub-grouping was concurrently performed in the original HIRAS database (Figure 2C) so that the previously calculated subgroup PPVs could be applied to the nationwide database. The PPVs of subgroups abc, bc, ac, and c, which were considered most likely to be true SLE patients, were 0.90, 0.76, 0.36, and 0.57, respectively. The prevalence of this algorithm was estimated using the following equation:

$$\begin{array}{l} Presumed\;number\;of\;SLE\;patients\\ =7842*\alpha +1672*\beta +260*\gamma +3878*\delta \end{array}$$

where α is PPV of group abc calculated from one institute, β is PPV of group bc, γ is PPV of group ac, and δ is PPV of group c. With subgroup analysis, estimated number of SLE patients was 10 633. Estimated number of SLE patients is about 10% more than other two methods.

**Figure 4.**
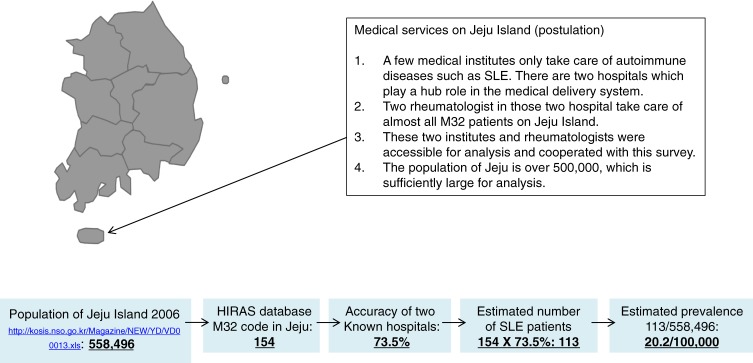
Calculation of the prevalence of SLE among the population of the isolated Jeju Island. Estimated SLE prevalence was again tested by the validation of M32-coded patients on the basis of the local island population. Local population based-estimation of SLE prevalence corresponded closely to the prevalence estimated from the nationwide database deduced by three different analyses.

Although prevalence was estimated by three different methods, the results were similar (Table). The total number of SLE patients was between 9000 and 11 000, and the prevalence of SLE was estimated to be 18.8–21.7 per 100 000 people in South Korea. Furthermore, after estimating SLE prevalence via three different methods, these estimates were compared to local SLE prevalence. Prevalence of SLE on the isolated Jeju Island was also calculated as 20.2 per 100 000 people, a similar estimate to the three shown in Table (Figure 4).

## DISCUSSION

This study is the first to estimate the prevalence of SLE in South Korea. Until now, most of the data regarding SLE prevalence have been based on surveys of western countries,^3^^,^^18^^–^^20^ and relatively old ones at that, although there are a few North American and European studies that have published data on unselected populations in the last 10 years.^6^^,^^7^ Results of a previous analysis estimated the prevalence of SLE to be about 14.6–50.8 per 100 000 people. In the recent US study of unselected populations, those cases were obtained from clinical records. Those studies estimated the prevalence to range from 78.5 (95% CI 59.0–98.0) per 100 000 people^21^ to 124 (95% CI 40.0–289.0) per 100 000 people.^22^

Our estimated prevalence, which was calculated using a single source of government data, was about 20 per 100 000 people. Although there may be ethnic differences in SLE prevalence between Korea and western countries, estimation by administrative data alone may not be a very sensitive means of identifying cases. Our methods would have underestimated SLE prevalence in South Korea if, for example, patients did not seek SLE-related medical care over the three consecutive years on which our prevalence study was focused. Though rare, it is likely to result in the under-ascertainment of milder forms of SLE. Omission of SLE records is also possible, especially when the medical care is primarily focused on diseases other than SLE. In a study using US Medicare data, Katz and colleagues compared diagnosis of SLE appearing in physician claims data with those appearing in medical records, which estimated the sensitivity of claims data to be 85% (95% CI 73%–97%).^14^ While the present study focused only on the billing data of rheumatologists, a more recent study by Nightingale and colleagues discussed the prevalence of SLE according to study duration.^23^ Their report pointed out that the likelihood of detecting SLE cases increased in proportion to the length of a patient’s contribution to the observational study, claiming that the incremental increase in SLE prevalence over time was, in fact, not a true outbreak of SLE, but an observational artifact.^14^

Recently, we asked the HIRA official statistical department of the South Korean government to provide the prevalence of SLE-coded patients who were diagnosed after 2009. HIRAS received 16 585 insurance claims reported with M32-coded cases in 2010. There was an annual increase of about 600–800 new cases from 2004 to 2006, while our final prevalence was 10 615 in 2006. If this annual increase continued to 2010, 4000–5000 new cases could be added to the 2006 prevalence data. Extrapolating these increases from the 2006 data resulted in similar numbers of patient cases reported in 2010 (16 585) and patients estimated from the 2006 database (around 15 000).

The annual increase of M32-coded patients in Figure 3A has several possible explanations: 1) decreased mortality or improvements in diagnostic methods might have led to an increase in new SLE cases; 2) increased numbers of rheumatologists might have enabled the diagnosis of SLE in previously underserved rural areas; or 3) environmental factors may have increased the actual incidence. The annual increase was seen primarily in patients aged 20–60 years, but was not prominent in groups less than 19 years old or more than 70 years old.

In observational studies, particularly when using administrative databases to identify cohorts of SLE patients, careful contemplation of the data source and consideration of alternative algorithmic definitions of the disease are imperative. The point estimates of prevalence can differ considerably depending on which approach is adopted for SLE detection, hampering determination as to whether or not one approach has greater validity than another. For this reason, we adopted three different algorithms to minimize over- or under-estimation of the true prevalence and obtained similar values in all attempted approaches.

Another strength of our study is that the presented prevalence incorporates the most widespread data available in South Korea to date. The distribution of our survey was extended to include many institutes in rural areas, where previous statistical investigations have omitted collection of data due to the distance and lack of resources. The rationale for our extensive survey was based on the fact that: (1) relatively few medical institutes provide specialized SLE care; (2) relatively few rheumatologists are in charge of SLE patients; and (3) despite the low number of specialized health care providers, the population of an isolated area should be sufficiently large for a valid analysis.

After calculating SLE prevalence via three different methods, we sought to verify whether or not these estimates are applicable to local prevalence. Our estimated prevalence in the isolated area of Jeju Island was 20.2 out of 100 000 people, which was similar to the results reported for national prevalence (Figure 4).

In summary, this study is the first attempt to estimate the SLE prevalence in South Korea, finding a prevalence of SLE in 2006 of 18.8–21.7 per 100 000 people. This study constructed three feasible algorithms to ascertain SLE cases in administrative data and validated these algorithms with a peer review process. Administrative databases may be a useful source of information in observational studies of patients with SLE. Since no method of case ascertainment can be absolutely accurate, there obviously is a risk of error in administrative data, which may have contributed to the variation of estimates in our study. Further research is needed to develop a disease model and an algorithm for the accurate estimation of the prevalence of rare autoimmune diseases.
